# Supplementary material for: Low prevalence of relative age effects in Luxembourg’s male and female youth football
Source: PLoS One. 2022 Aug 23;17(8):e0273019. doi: 10.1371/journal.pone.0273019 (PMC9398004; doi:10.1371/journal.pone.0273019)
Supplement: Attachment — Submitted filename: answers to reviewer comments.docx [file pone.0273019.s002.docx]

Top of Form

Thank you for your careful consideration of our manuscript and the positive feedback. All comments by the reviewers have been addressed, with corresponding changes made directly to the manuscript where appropriate. Accordingly, we send you our revised manuscript with all the changes made during the revision process in coloured text/track changes. In this rebuttal letter you will find our detailed point-by-point response to the comments raised by the reviewers about our manuscript.

We would like to take this opportunity to express our sincere thanks to the reviewers who identified areas of our manuscript in need of corrections or modifications. We would also like to thank you for allowing us to resubmit a revised copy of the manuscript.

**5. Review Comments to the Author

Reviewer #1:** The authors' research question is well defined and supported in the statistical analyses presented.

The study does not prove the identified cause for the inexistence of RAE in Luxembourgish youth football. It only proves the non-existence of RAE, not the cause, as suggested by the title of the article: "Luxembourg’s open-door player selection policy reduces the risk of a relative age effect in youth football".

To make the title of the paper truly match the results it should be something like: " Luxembourg's youth football teams exhibit a negligible relative age effect".

Thank you, this is an important point. We agree that this study does not identify a clear cause (open-door policy) ⬄ effect (low RAE) effect although it may be reasonable to assume that it contributes to the low RAE prevalence. Therefore, the title has been rephrased. The short title has been adjusted accordingly.

The only suggestion I would make to the paper is to introduce 1 or 2 charts showing the results, as they make the paper more appealing.

Thank you. Two charts (Figure 1 and Figure 2) have been included in the manuscript illustrating the results of the overall Luxembourgish soccer population, the national players as well as the top and bottom tear teams. To further account for the comments of reviewer 2, we decided to show the results for the male and female players where possible.

**Reviewer #2:** Thank you for the opportunity to review this article. The paper addresses a novel under-researched are. However, there are some questions that need to be addressed to the manuscript.


KEYWORDS
Please, avoid using the same words of the title in the keywords.

Thank you, this has been corrected

INTRODUCTION
The introduction section is clear and well-written. However, it is necessary to elaborate a paragraph explaining in depth the relationship between RAE and performance in both genders.

Thank you for mentioning this point. An additional paragraph on the RAE in female football has been added to the introduction section (ll. 70-81).


MATERIAL AND METHODS
Name “under” before. (line 97)

This has been changed

Add the year of the Declaration of Helsinki (line 104)

The year of the last amendment in 2013 has been added.

Are these years correct? (line 141)

Thank you there was a typo which has been corrected (2000-2007).

“p” in italic (line 157)

Thank you. This has been changed


DISCUSSION
The discussion is clear and consistent. Great job! However, where the discussion of female results? Please, include it.

Thank you. Similar to the introduction, an additional section has been added discussing the results of the female athletes for the whole female player population (ll.267-284) as well as for the top and bottom teams (ll. 306-317).

Relative age effects need to be abbreviated. (line 207)

This has been changed.

Add population (line 233)

Additional information on the overall Luxembourgish population size in the age groups of interest have been added to the manuscript (ll. 263-264).

What will happen to these talented players if they cannot enhance their performance for this reason? Maybe, they would not be able to be successful in the future? This idea is not good for the reader... Please, discuss it. (line 233-234)

We agree that the instead of negative consequences of the open-door policy, a more positive attitude would be preferable to avoid the impression that the RAE benefits performance. This part has been rephrased on now focusses on the chances of giving late born players the chance to continue their professional career despite physiological disadvantages during maturation (ll. 267-284).
